# Extensive citrullination of human serum albumin is physiological and not inherently immunogenic in rheumatoid arthritis

**DOI:** 10.1016/j.jbc.2025.110438

**Published:** 2025-07-01

**Authors:** Cecilia Mustelin, Archana Einstein, Xiaoxing Wang, Farheen Shaikh, Tomas Mustelin

**Affiliations:** Division of Rheumatology, Department of Medicine, University of Washington, Seattle, Washington, USA

**Keywords:** albumin, citrullination, rheumatoid arthritis, anti-citrullinated protein antibodies, protein arginine deiminase, thyroxin

## Abstract

Autoantibodies against citrullinated proteins are diagnostic of rheumatoid arthritis (RA), a chronic and systemic autoimmune condition that affects synovial joints. Proteomic studies have revealed that human serum albumin is among the proteins that are citrullinated in RA. Anti-citrullinated protein antibodies reacting with albumin have also been reported. Here, we show that albumin is citrullinated at 11 arginine residues in the blood of both RA patients and, surprisingly, in healthy donors and to a very similar stoichiometry, albeit with some subtle differences. Albumin citrullination exhibited slightly different patterns in synovial fluid from RA patients compared to RA serum-derived albumin, although overall stoichiometry was similar. Incubation of albumin with live neutrophils or recombinant protein arginine deiminases 2 or 4 at 37 °C resulted in its rapid citrullination at multiple sites. Albumin citrullination reduced thyroxin binding *in vitro*. IgG antibodies in the serum of RA patients and healthy donors displayed comparable reactivities to physiologically citrullinated albumin. Similarly, citrullinated peptides corresponding to 14 citrullination sites were not significantly better recognized by IgG in serum from 86 RA patients than from healthy controls, and surprisingly some were even recognized to a lesser degree in RA. The very few RA patient antibodies exceeding the 95th percentile of the signal in healthy donors may simply represent weak cross-reactivity of antibodies against unrelated citrullinated antigens. Our findings reveal that albumin citrullination is likely physiological and of little interest to the immune system in RA patients, presumably because of persisting immunological tolerance. We discuss potential physiological functions of albumin citrullination.

Rheumatoid arthritis (RA) is a chronic and systemic autoimmune disorder that primarily affects synovial joints (1, 2), notably the proximal finger joints, wrists, elbows, feet and knees. In addition to joint pain, stiffness, and swelling, patients often experience fatigue, anemia, fever, rheumatic nodules, and interstitial lung disease. Although many treatment options ranging from blockers of tumor necrosis factor α, interleukin-6, Janus kinases, as well as T cell–targeted therapies and B cell depletion are now are available (3), many RA patients continue to suffer from insufficiently controlled disease.

An important clue to the pathogenesis of RA was published 25 years ago by Shellekens *et al.* (4), who discovered that sera from a high proportion of RA patients contained antibodies reactive with cyclic citrullinated peptides (CCPs), now commonly referred to as anti-citrullinated protein antibodies (ACPAs). Due to their high specificity for RA (4, 5) and their presence even before symptom onset (6), the CCP test now forms a cornerstone of the diagnosis of RA, particularly in distinguishing it from other forms of arthritis (7). ACPA-positive patients usually also test positive for rheumatoid factor and are referred to as having “seropositive” RA. These patients typically have the classical form of RA with the well-recognized course, range of symptoms, and prognosis, and they benefit from a range of commonly used therapeutics. In contrast, patients with seronegative disease (CCP- and rheumatoid factor-) have a more variable disease presentation, likely representing several distinct disease entities.

The specificity of ACPAs for RA indicates that protein citrullination must play a unique role in this disease. While citrullination is a posttranslational modification involved in many physiological processes (8), RA is characterized by qualitatively and quantitatively abnormal protein citrullination catalyzed by protein arginine deiminases (PADs) PAD2 and PAD4 (9). Protein citrullination entails the conversion of a positively charged arginine (Arg, R) residue to a neutral citrulline (8), which may have a profound impact on the structure and function of the targeted protein (10). We have proposed that nonphysiological citrullination creates “neoantigens” that appear indistinguishable from pathogen-derived antigens to the immune system; central T cell tolerance simply does not exist toward novel citrullinated peptides presented on major histocompatibility antigens (11). This results in T and B cell immunity (12) against an increasing repertoire of citrullinated epitopes over the course of the disease (13). Seropositive RA patients with the highest concentration of PADs and citrullinated proteins in their synovial tissue also had the highest degree of tissue damage (14).

Human serum albumin (HSA, here referred to as just “albumin”) is the most abundant extracellular protein at 3.4 to 5.5 mg/ml in blood and extracellular fluid and constitutes approximately 56% by weight of the synovial fluid in healthy people. Albumin plays major roles in synovial lubrication, colloidal osmotic pressure (15), ligand binding and transport, and antioxidant activity (16). Albumin also participates in inflammatory processes where it modulates immune responses through Toll-like receptor signaling (17), increases glutathione in lymphocytes, and inhibits tumor necrosis factor α–mediated NF-κB activation associated with oxidative stress and apoptosis (18). The importance of albumin is well illustrated by the symptoms of even mild hypoalbuminemia (caused by malnutrition, severe anorexia, sepsis, liver cirrhosis, nephrotic syndrome, or protein-losing enteropathy), which include altered fluid distribution between extracellular and intracellular compartments, circulatory disturbances, edema, ascites, and effusions around internal organs.

Of relevance to the present study, albumin is often lower in the circulation of RA patients (19) and even lower in synovial fluid at sites of active inflammation (20). These findings may be explained by the increased metabolic demand at sites of inflammation in the RA synovium, which increases vascular permeability by up to 6-fold (21) and promotes albumin uptake by synovial cells. Albuminuria, or increased urinary excretion of albumin as a result of enhanced vascular permeability, is a common finding associated with disease duration (22), arterial stiffness (23), overall systemic inflammation (24), and increased mortality in RA (25). Decreased serum levels of albumin paradoxically increase its half-life, potentially by as much as 50 to 100 days (26), allowing the protein to accumulate increasing numbers of posttranslational modifications as it stays in circulation.

The current study seeks to better characterize the repertoire and stoichiometry of albumin citrullination sites in RA by mass spectrometry and to assess ACPA reactivity at each site compared with healthy control (HC) and systemic lupus erythematosus patients as a disease control. As albumin is the most abundant protein in blood and extracellular fluid, even a modest binding of autoantibodies to citrullinated albumin could have clinically relevant consequences. However, our findings revealed that albumin is also citrullinated similarly in healthy donors and that the immune system in RA patients is not particularly interested in citrullinated albumin, presumably because preexisting immunological tolerance persists in RA patients.

## Results

### RA patients and CCP status

The RA patients included in this study encompassed n = 86 serum samples. This cohort represents a typical medical center RA patient population (Table 1) with an average age of 53.3 ± 14.0, 73.3% female, a range of disease activity levels from high (14.7%) to remission (27.9%), and medications ranging from methotrexate and other disease-modifying antirheumatic drugs to corticosteroids (18.6%), and biologics (64%). To verify the patient records of CCP status, we used the IMMUNOSCAN CCPlus kit to measure their IgG ACPA and found that 50 (59.5%) were positive (Table 1). Only two patients yielded different results compared to their historical medical records. HCs were CCP negative.

**Table 1:** Patient demographics, serological, and disease data

Age^a^	53.3 ± 14.0 years (range: 22–82)
Sex	63 (73.3%) females
Anti-citrullinated protein Ab	CCP+ 50 (59.5%)
	CCP− 34 (40.5%)
Rheumatoid factor	RF+ 49 (58.3%)
	RF− 35 (41.7%)
Clinical disease activity index (CDAI):	High (≥22): 10 (14.7%)
(n = 68)	Low–moderate (2.8–22): 39 (57.4%)
	Remission (≤2.8): 19 (27.9%)
Disease duration in years (n = 84):	10.8 ± 9.8 years
Treatment classes^b^:	DMARD^c^: 66 (76.7%)
	Biologics: 55 (64.0%)
	Corticosteroids^d^: 16 (18.6%)
	Other^e^: 5 (5.8%)

CCP, cyclic citrullinated peptide.

a Average ± standard deviation (range).

b Note that many patients were taking more than one class of drugs.

c DMARD = disease-modifying antirheumatic drugs (methotrexate, leflunomide, hydroxychloroquine, sulfasalazine).

d 12 patients reported taking regularly, the rest were taking as needed.

e Other medications included immunosuppressants (n = 3), non-steroidal anti-inflammatory drugs (n = 1), and opioids (n = 1).

### Identification of citrullinated Arg residues in albumin

**Figure 1:**
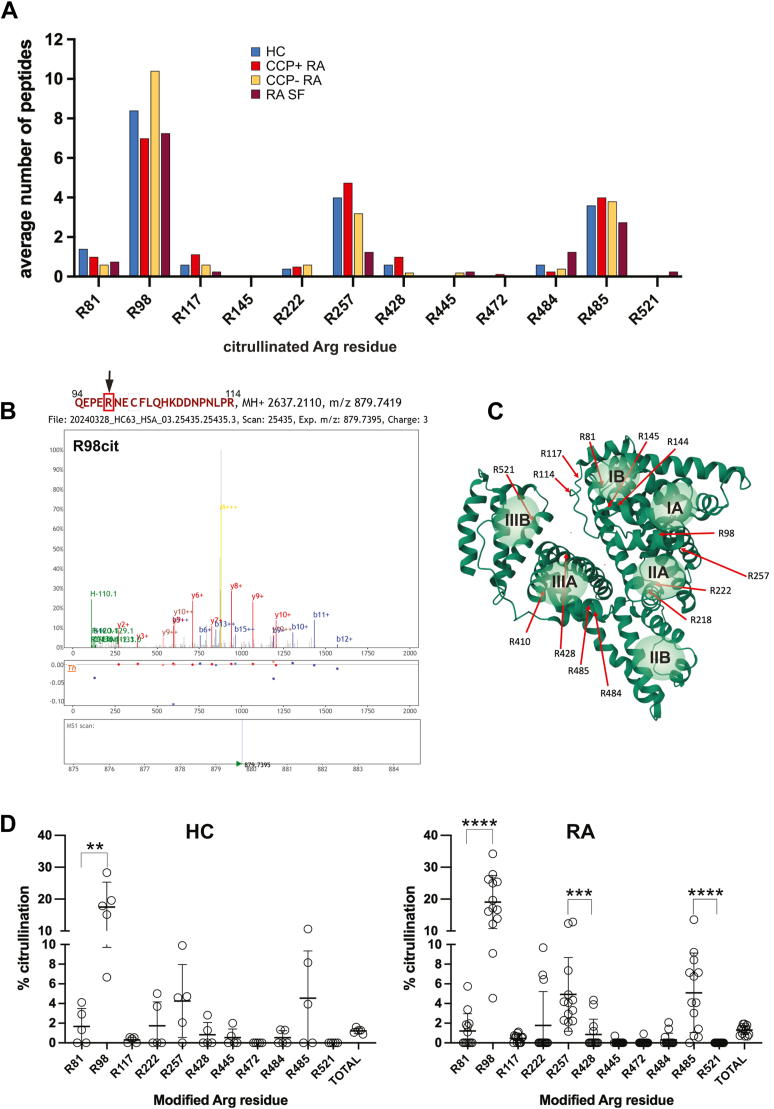
**Summary of mass spectrometry analyses to identify citrullinated Arg residues in albumin.***A*, identified sites by the average number of peptides detected in LC-MS/MS experiments in healthy controls (HCs, *blue bars*), CCP-positive RA patients (*red bars*), CCP negative RA patients (*golden bars*), and in synovial fluid from an RA patient (*purple bars*). *B*, mass spectrum for the peptide containing citrullinated R98. *C*, location of the modified Arg residues in the three-dimensional structure of albumin. The six ligand binding pockets are indicated as *pale green spheres*. *D*, stoichiometry of Arg citrullination in albumin from healthy donors (HC; n = 5) and RA patients (n = 13). Statistical significance was assessed using the Mann–Whitney *U* test. ∗∗, *p* < 0.01 and ∗∗∗∗, *p* < 0.001. CCP, cyclic citrullinated peptide; RA, rheumatoid arthritis.

Mature albumin consists of 585 amino acid residues, 24 of which are Arg. When albumin in serum or synovial fluid from 13 RA patients (n = 13 serum, n = 2 synovial fluid), two non-RA inflammatory controls (n = 1 juvenile idiopathic arthritis, n = 1 an otherwise healthy person who underwent knee surgery for a torn meniscus), five healthy blood donors, obtained commercially as a purified protein, or derived from heat-inactivated serum was digested to completion by trypsin and analyzed by LC-MS/MS, we detected numerous spectral counts corresponding to peptides (hereafter referred to as just “number of peptides”) with an Arg converted to citrulline and that met the quality criteria of high-confidence identification. The modified Arg corresponded to R81, R98, R117, R222, R257, R428, R445, R472, R484, R485, and R521 (Fig. 1*A*). Additional sites only meeting somewhat less stringent criteria were observed in some samples. A representative mass spectrum is shown in Figure 1*B* and six more in Fig. S1. The locations of the modified residues in the three-dimensional structure of albumin are shown in Figure 1*C*.

The number of peptides containing a given modified Arg residue constitutes, at best, a semiquantitative estimate of abundance allowing for a comparison of each site between samples (but not between sites). By this measure, albumin from RA synovial fluid was citrullinated to a similar stoichiometry as albumin in blood from RA patients albeit with some nonsignificant differences. Three different preparations (see Experimental procedures) of the one RA synovial fluid sample resulted in similar citrullination profiles, but with marginally higher overall citrullination in the acetone precipitation sample (1.13% *versus* 0.75%).

Surprisingly, albumin from healthy donor sera contained the same pattern of citrullination as albumin from the blood of RA patients (Fig. 1*A* and Table 2). The only Arg residues citrullinated in RA but not in healthy donors were R445, R472, and R521, which were represented by only a single peptide in either one or two samples.

**Table 2:** Relative abundance^a^ of citrullinated albumin peptides in sera and synovial fluid from healthy donors and RA patients

Site^b^	RA: Serum (n = 13)	HC: Serum (n = 5)	RA: SF (n = 4)^c^	Non-RA^d^: SF (n = 2)
R81	0.8 ± 1.4 (0–5)	1.4 ± 1.5 (0–3)	0.8 ± 1.0 (0–2)	0.5 ± 0.7 (0–1)
R98	8.3 ± 4.8 (2–16)	8.4 ± 4.7 (3–15)	7.3 ± 3.1 (3–10)	3.5 ± 2.1 (2–5)
R114	0	0	0	0
R117	0.9 ± 0.9 (0–2)	0.6 ± 0.5 (0–1)	0.3 ± 0.5 (0–1)	0
R144	0	0	0	0
R145	0	0	0	0
R218	0	0	0	0
R222	0.5 ± 1.1 (0–3)	0.4 ± 0.5 (0–1)	0	0
R257	4.1 ± 3.1 (1–11)	4.0 ± 3.7 (0–10)	1.3 ± 1.0 (0–2)	2.0 ± 1.4 (1–3)
R410	0	0	0	0
R428	0.7 ± 1.3 (0–4)	0.6 ± 0.9 (0–2)	0	0.5 ± 0.7 (0–1)
R445	0.1 ± 0.3 (0–1)	0	0.3 ± 0.5 (0–1)	0
R472	0.1 ± 0.3 (0–1)	0	0	0
R484	0.3 ± 0.6 (0–2)	0.6 ± 0.9 (0–2)	1.3 ± 1.9 (0–4)	0
R485	3.9 ± 2.7 (0–8)	3.6 ± 3.5 (0–8)	2.8 ± 2.4 (1–6)	2.0 ± 1.4 (1–3)
R521	0	0	0.3 ± 0.5 (0–1)	0
Total	19.8 ± 7.3 (8–32)	19.6 ± 4.7 (16–26)	13.8 ± 7.1 (8–24)	8.5 ± 3.5 (6–11)

HC, healthy control; RA, rheumatoid arthritis; SF, synovial fluid.

a Expressed as the average total spectral counts for each citrullinated Arg ± SD (range).

b Only Arg residues corresponding to the peptides used in the ELISAs are included.

c Three different preparations of the same RA SF sample (IgG-depletion, acetone precipitation, pellet) and one additional RA SF sample.

d One healthy donor and one juvenile idopathic arthritis.

Dividing the RA patients into two groups by CCP status (4 CCP-, 9 CCP+) showed that sera from both groups had very similar abundances of citrullinated peptides. A minor and statistically nonsignificant higher citrullination at R81, but lower at R98, was observed in CCP+ patients than CCP- patients (Fig. 1*A*). We conclude that any possible CCP-related differences are negligible compared to the individual variation between patients.

### Stoichiometry of albumin citrullination in RA and healthy donors

The pronounced variability in citrullinated peptide counts between samples complicated the interpretation of citrullination based solely on spectral counts. Therefore, we calculated an independent normalized estimate of citrullination stoichiometry for each modified Arg from the number of peptides with citrulline as a percent of all peptides containing each specific Arg, whether citrullinated or not (Fig. 1*D* and Table 3).

**Table 3:** Stoichiometry of albumin citrullination^a^ in sera and synovial fluid from healthy donors and RA patients

Site^b^	RA: Serum (n = 13)	HC: Serum (n = 5)	RA: SF (n = 4)^c^	Non-RA^d^: SF (n = 2)
R81^e^	1.21 ± 1.75 (0–5.75)	1.67 ± 1.82 (0–4.11)	1.36 ± 1.74 (0–3.64)	1.00 ± 1.41 (0–2.00)
R98	19.07 ± 8.34 (4.55–34.21)	17.51 ± 7.81 (6.67–28.3)	24.38 ± 6.59 (17.65–33.33)	22.40 ± 9.92 (15.38–29.41)
R114	0	0	0	0
R117	0.45 ± 0.40 (0–1.12)	0.30 ± 0.28 (0–0.55)	0.05 ± 0.10 (0–0.20)	0
R144	0	0	0	0
R145	0	0	0	0
R218	0	0	0	0
R222	1.77 ± 3.44 (0–9.68)	1.74 ± 2.43 (0–5.00)	0	0
R257	4.93 ± 3.74 (1.15–12.82)	4.25 ± 3.71 (0–9.90)	0.28 ± 0.25 (0–0.50)	0.44 ± 0.11 (0.36–0.52)
R410	0	0	0	0
R428	0.85 ± 1.55 (0–4.35)	0.82 ± 1.25 (0–2.82)	0	0
R445	0.05 ± 0.19 (0–0.70)	0	0.31 ± 0.63 (0–1.25)	0
R472	0.07 ± 0.25 (0–0.92)	0	0	1.92 ± 2.72 (0–3.85)
R484	0.31 ± 0.67 (0–2.08)	0.53 ± 0.72 (0–1.34)	1.25 ± 2.03 (0–4.26)	0
R485	5.09 ± 4.04 (0–9.21)	4.55 ± 4.79 (0–10.64)	4.07 ± 3.26 (0.74–7.32)	2.44 ± 2.44 (0.72–4.17)
R521	0	0	25.00 ± 50.00 (0–100.00)	0
Total	1.30 ± 0.46 (0.68–1.93)	1.22 ± 0.27 (0.88–1.29)	0.71 ± 0.33 (0.32–1.13)	0.47 ± 0.35 (0.22–0.72)

HC, healthy control; RA, rheumatoid arthritis; SF, synovial fluid.

a Citrullinated peptides as percent of all peptides covering each site ± SD (range).

b Only Arg residues corresponding to the peptides used in the ELISAs are included.

c Three different preparations of the same RA SF sample (IgG-depletion, acetone precipitation, pellet) and one additional RA SF sample.

d One healthy donor and one juvenile idopathic arthritis.

e None of the comparisons between HC and RA were statistically significant using the Wilcoxon signed-rank test.

For serum samples, the highest stoichiometry was observed at R98, with 19.07 ± 8.34% (range 4.55–34.21%) in RA patients (n = 13) and 17.51 ± 7.81% (6.67–28.3%) in healthy donors (Table 3). R485 was the second most citrullinated site, with 5.09 ± 4.04% (0–9.21%) in RA and 4.55 ± 4.79% (0–10.64%) in healthy donors, followed by R257 with 4.93 ± 3.74% (1.15–12.82%) in RA and 4.25 ± 3.71% (0–9.90%) in healthy donors. Other citrullinated sites included R81 and R222 with comparable citrullination stoichiometry between groups. All other sites were citrullinated to less than 1%. Overall albumin citrullination was 1.30 ± 0.46% (0.68–1.93%) in RA and 1.22 ± 0.27% (0.88–1.29%) in healthy donors.

Albumin citrullination patterns in RA synovial fluid samples were similar to that in serum with some minor differences: R81, R98, R484, and R521 appeared more citrullinated, and R257 less, in synovial fluid compared to serum. Albumin in non-RA synovial fluid also appeared to be somewhat less citrullinated at R81, R98, R484, R485, and R521. However, as our small sample size precludes statistical testing, we view these as nonsignificant.

These data agree with Figure 1*A* but give a better view of the state of albumin citrullination, which clearly is very similar in healthy donors *versus* RA patients.

### Location of the citrullinated Arg residues in albumin

To assess the accessibility of the targeted Arg side chains in the three-dimensional structure of albumin, we located the modified R residues in the crystal structure of albumin (Fig. 1*B*, PDB ID: 1AO6). Many of the modified resides are exposed or tangential to the surface and, hence, predicted to be readily accessible to PAD enzymes (Table 4). However, there are some notable exceptions. Specifically, the most prominently citrullinated residue, R98, has a solvent-accessible surface area of only 18.65 Å^2^ calculated by PyMol (https://www.pymol.org), yet approximately 20% of circulating albumin is citrullinated at this site. On the other hand, the Arg with the largest solvent-accessible surface area of 185.13 Å^2^, R114, was not citrullinated in this study. This suggests that albumin might undergo conformational changes while in circulation or bound to ligands that open up less accessible sites to the PAD enzymes, while perhaps decreasing accessibility of other sites. It is also possible that citrullination of one site may induce conformational changes that either facilitate or hinder subsequent citrullination of other sites.

**Table 4:** Arg side chain exposure and substrate preference by PADs

Site	SASA^a^	% PAD2 citrullination^b^	% PAD4 citrullination^b^
R10	28.82 Å^2^	0%	0%
R81	177.91 Å^2^	18.92%	89.34%
R98	18.65 Å^2^	43.48%	23.53%
R114	185.13 Å^2^	1.53%	0.44%
R117	81.94 Å^2^	2.23%	0%
R144	24.73 Å^2^	0%	0%
R145	54.15 Å^2^	2.78%	0%
R160	71.40 Å^2^	0%	0%
R186	131.99 Å^2^	0%	0%
R197	28.38 Å^2^	0%	0%
R209	126.19 Å^2^	0%	0%
R218	43.93 Å^2^	25.0%	0%
R222	33.88 Å^2^	0%	0%
R257	23.19 Å^2^	0.82%	0%
R336	27.34 Å^2^	0%	0%
R337	41.68 Å^2^	0%	0%
R348	16.19 Å^2^	0%	0%
R410	74.43 Å^2^	16.67%	0%
R428	44.07 Å^2^	25.0%	18.75%
R445	50.13 Å^2^	0%	0%
R472	55.07 Å^2^	57.14%	11.11%
R484	21.39 Å^2^	3.88%	0.65%
R485	11.15 Å^2^	2.91%	0.65%
R521	13.74 Å^2^	0%	0%

HSA, human serum albumin; PAD, protein arginine deiminase.

a SASA, solvent accessible surface area, calculated from the crystal structure of human serum albumin (PDB ID: 1AO6).

b Citrullination in AB+ serum-derived HSA treated with PAD2 or PAD4.

The modified residues are all close to each of the six ligand binding pockets of albumin: R81 and R98 by pocket IA, R117, R144, and R145 by pocket IB, R222, and R257 by pocket IIA, R428, R445, and R485 by pocket IIIA, and R521 by pocket IIIB (Fig. 1*B*). Thus, it is conceivable that citrullination may affect albumin’s ability to interact with certain ligands.

**Figure 2:**
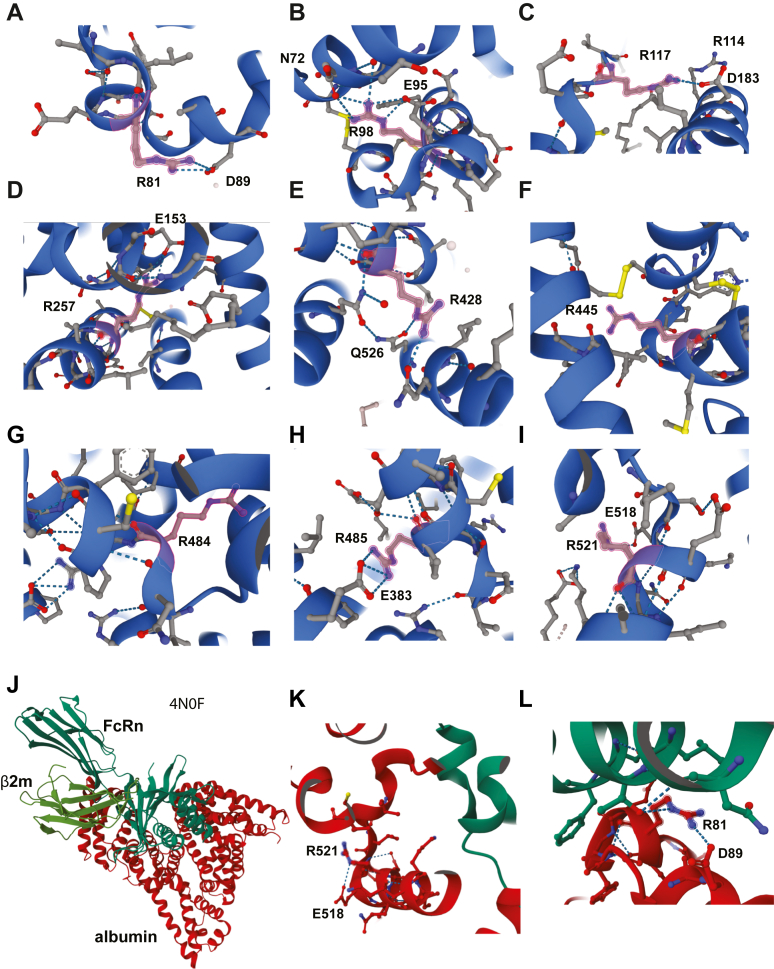
**The location and interactions of Arg found to be citrullinated in albumin.** Location of the modified Arg residues in the three-dimensional structure of albumin. *A*, R81. *B*, R98. *C*, R117. *D*, R257. *E*, R428. *F*, 445. *G*, R484. *H*, R485. *I*, R521. *J*, the crystal structure (4NF09) of albumin (*red*) bound to the FcRn (*dark green*), including β2 microglobulin (*lighter green*), in the same orientation as in Figure 1*D*. *K*, close-up of R521 near the interface with FcRn (*green*). *L*, same for R81. FcRn, neonatal Fc receptor.

A closer inspection (Fig. 2) revealed that the guanidine groups of R81 (Fig. 2*A*), R98 (Fig. 2*B*), R117 (Fig. 2*C*), R257 (Fig. 2*D*), R428 (Fig. 2*E*), and R485 (Fig. 2*H*), and R521 (Fig. 2*I*), but not R445 (Fig. 2*F*) or R484 (Fig. 2*G*), make ionic interactions with nearby residues likely stabilizing the local structure. The removal of the charge of these resides during citrullination will eliminate these bonds resulting in altered connections between structural elements within albumin, potentially causing conformational changes of parts of the protein.

The crystal structure (4NF09) of albumin bound to the neonatal Fc receptor (FcRn), a receptor critical for albumin’s extraordinarily long-half life by salvaging it from degradation along with IgG (27) (Fig. 2*J*) revealed that several of the citrullinated Arg residues in albumin are located near the interface with FcRn, most notably R81 (Fig. 2*L*), R114, R428, and R521 (Fig. 2*K*). Additionally, R81 makes a direct hydrogen bond with T150 of FcRn and an intramolecular hydrogen bond with nearby D89, which likely contributes to the stability of the albumin–FcRn complex. R114, R428, and R521 do not directly contact FcRn, but mutagenesis studies of nearby residues have demonstrated their important contribution to the FcRn interface (28). Hence, their citrullination may affect albumin binding to FcRn, as suggested by the 50% reduction in FcRn binding by the R81A mutation (29) and the effects of several mutations close to R521: K519E (30, 31) and I523G (31). However, we cannot exclude the possibility that other sites may also contribute to interactions with FcRn through their intramolecular interactions with neighboring residues and overall structural contributions.

### Citrullination of albumin by PAD2, PAD4, and live neutrophils

Albumin is synthesized by the liver, in which, to the best of our knowledge, citrullination of secreted proteins has not been reported. Furthermore, the intracellular pathway through the Golgi apparatus and secretory vesicles used for albumin export from hepatocytes does not intersect with the known cytosolic or nuclear locations of PAD enzymes. Hence, albumin is likely citrullinated after release from the liver into the extracellular space, perhaps in circulation or in the joint of RA patients. Thus, we first asked whether PAD2 and PAD4 citrullinated albumin *in vitro*, and whether they did so to different degrees or at different sites. Next, we asked if neutrophils, which expose catalytically highly active PAD4 on their surface and secrete PAD2 (32), can also citrullinate albumin.

**Figure 3:**
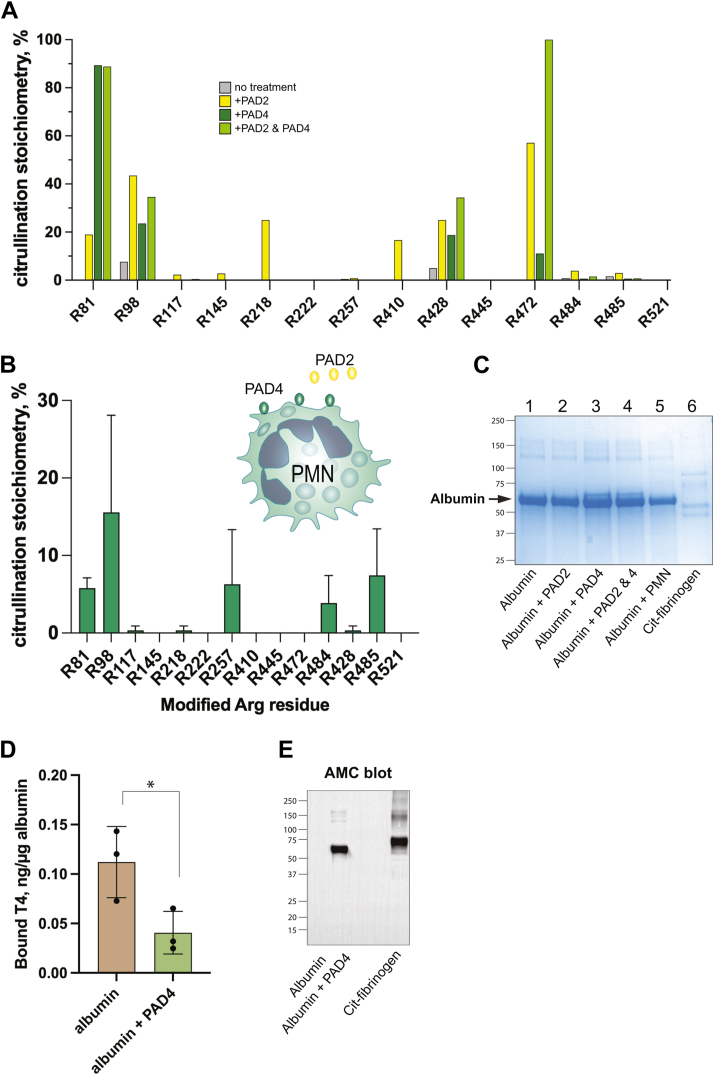
**Citrullination of albumin by PAD2, PAD4, and live neutrophils.***A*, stoichiometry of citrullination of albumin at all sites after treatment without (*gray*) or with PAD2 (*yellow*), PAD4 (*dark green*), or both enzymes (*pale green*). *B*, Arg residues in albumin citrullinated by live neutrophils. *C*, Coomassie *blue*–stained gel of albumin treated with PADs or neutrophils (PMN), as indicated. *D*, binding of thyroxin (T4) by albumin treated with (*green*) or without (*brown*) PAD4, as indicated. Statistical significance was assessed using the Mann–Whitney *U* test. *E*, anti-modified citrulline (AMC) of albumin treated with PAD4 with citrullinated fibrinogen as a positive control. PAD, protein arginine deiminase.

Indeed, incubation of human serum-derived 96% purified albumin with PAD2, PAD4, both together, or RA neutrophils at 37 °C resulted in increased citrullination at multiple sites. This preparation of albumin had lower citrullination at R98 than albumin in RA or healthy donor blood or synovial fluid. We also tested heat-inactivated normal human AB+ serum (depleted of IgG; (33, 34)), in which albumin was also citrullinated at multiple sites with a pattern similar to that of albumin in serum and synovial fluid (Fig. 3*A*).

PAD2 had slightly broader selectivity than PAD4 and citrullinated 11 sites (R81, R98, R117, R145, R218, R257, R410, R428, R472, R484, and R485), some of which were undetectable before the incubation. PAD2 increased R98 (+35.79%) and R472 (+57.14%) citrullination the most (Table 4). The increase in R98 citrullination was over 2-fold higher with PAD2 than PAD4 (+15.84%), which aligns with previous findings that neutrophils from healthy people secrete PAD2 at rest in the absence of stimulation (32).

PAD4 citrullinated fewer sites than PAD2 (R81, R98, R428, R472) and was particularly selective for R81, the citrullination of which increased to an 89.34% stoichiometry (Table 4). The degree of citrullination by PAD4 was lower at all other sites than PAD2. However, PAD4 increased overall albumin citrullination more than PAD2 (3.74% for PAD2 *versus* 13.70% for PAD4), suggesting that the high citrullination of R81 by PAD4 contributes substantially to overall albumin citrullination. Indeed, citrullinated R81 peptides compromised over 95% of all citrullinated peptides induced by PAD4. Curiously, the stoichiometry of R81 citrullination showed a moderate correlation with RA clinical disease activity index (r = 0.56, *p* = 0.0477) although there was no statistically significant difference between RA patients and HCs.

When PAD2 and PAD4 were incubated together with albumin, overall citrullination was slightly lower than with PAD4 alone (12.18%). Some sites were citrullinated to a higher degree (R428, R472), some to a lesser degree (R117, R485), some to a similar degree (R81, R98, R484), and some not at all despite being citrullinated by one or the other separately (R145, R218, R257, R410) (Fig. 3*B*). Figure 3*C* shows the total number of peptides in albumin before and after incubation with PAD4, a change from 8 to 229 citrullinated peptides.

In the presence of neutrophils, albumin gained a higher stoichiometry of citrullination at R81, R257, R484, and R485 (Fig. 3*B*). This pattern was not identical to the effects of recombinant PAD2, PAD4, or the combination, indicating that surface-exposed PAD4 and/or secreted PAD2 on live neutrophils may have catalytic properties that differ from bacterially expressed PAD enzymes. A Coomassie blue–stained gel verified that there were similar amounts of albumin in each sample (Fig. 3*C*).

### *In vitro* citrullinated albumin binds less thyroxin

One of albumin’s functions in circulation is to bind and transport metabolites, short-fatty acids, drugs, and hormones. To test if citrullination of albumin would affect its binding of thyroxin (T4), we treated 30 μg of purified albumin with or without 1 μg PAD4 for 30 min at 37 °C and then incubated it with T4 for 30 min. The mixture was then transferred to a spin column with a 10 kDa cut-off filter and given a 5 min centrifugation. The unbound and bound T4 was measured by a special ELISA kit for this purpose. These experiments showed that albumin citrullination decreased T4 binding to 36.3% of the untreated albumin (Fig. 3*D*).

### Anti-modified citrulline blot

The citrullination of albumin in this experiment was verified by an anti-modified citrulline immunoblot (Fig. 3*E*), in which a chemical modification of citrulline by diacetyl monoxime and antipyrine in a strong acid mixture (35) makes it reactive with a specific antibody. This assay is not very sensitive and untreated albumin remained undetected. As a positive control, we included citrullinated fibrinogen, in which all three subunits were recognized in the anti-modified citrulline (AMC) blot (Fig. 3*E*, last lane).

### IgG ACPA reacting with individual citrullination sites in albumin

**Figure 4:**
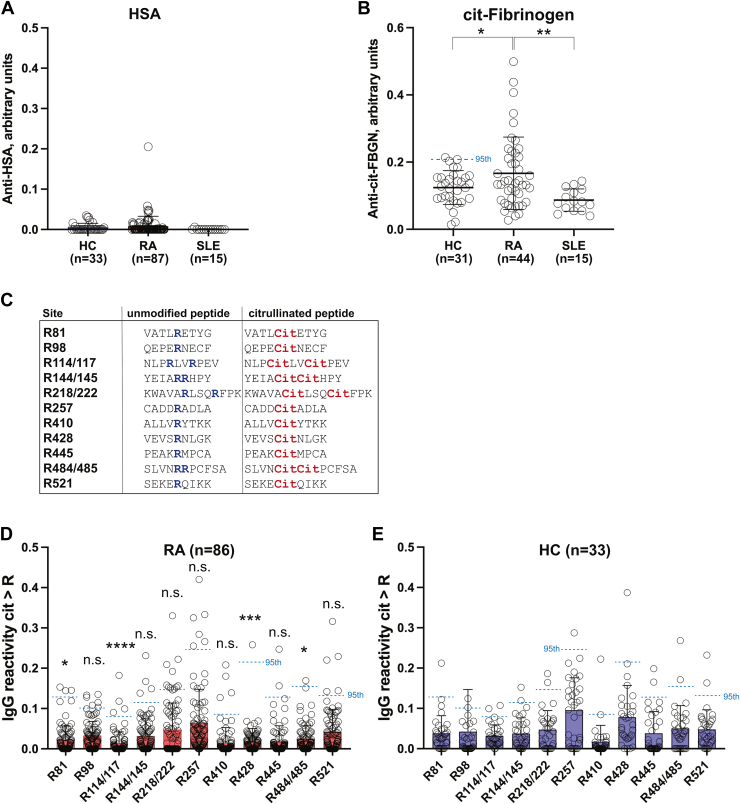
**ELISAs with intact albumin and citrullinated albumin peptides.***A*, detection by ELISA of IgG antibodies binding purified albumin. *B*, positive control ELISA with the same serum samples detecting IgG binding citrullinated fibrinogen. Statistical significance was assessed using the Mann–Whitney *U* test. *C*, sequence of all peptides used in ELISAs. The unmodified Arg residues are indicated in *blue* and the citrulline residues in *red*. *D*, IgG reactivity in RA sera (n = 86) against the indicated citrullinated peptides after subtraction of the reactivity against the unmodified peptide. *E*, the exact same ELISA using healthy control (HC) sera (n = 33). The 95th percentile of the HC distribution for each individual peptide is indicated as a *horizontal dotted line*. Statistical significance was assessed using the Mann–Whitney *U* test for reactivity for each peptide between HC and RA. Note that the statistically significant values all represent lower reactivity in RA than in HC. ∗, *p* < 0.5; ∗∗, *p* < 0.01; ∗∗∗, *p* < 0.005, and ∗∗∗∗, *p* < 0.001. RA, rheumatoid arthritis.

We reported a few years ago that approximately half of all RA patients have IgG autoantibodies that recognize *in vitro* citrullinated albumin (36), but not untreated albumin. However, we did not know at the time that untreated albumin is already citrullinated. We were also not able to conclude that citrullinated albumin was the primary target for these autoantibodies. Rather, they may represent cross-reacting ACPA. Figure 4*A* replicates our finding: an ELISA with untreated purified albumin (from Thermo Fisher Scientific), which we now know is citrullinated at multiple sites (albeit most of them at low stoichiometry), yielded no IgG binding at all in a majority of the healthy donor (n = 24), RA patient (n = 86), or SLE patient (n = 24) sera. Only one RA serum sample contained IgG with affinity for albumin well above the HCs. In contrast, the positive control citrullinated fibrinogen (Fig. 4*B*) was recognized by 13 patients above the 95th percentile of the healthy donor values, while IgG in SLE serum reacted even less than healthy donor IgG (Fig. 4*B*). Even in aggregate, the RA reactivity to citrullinated fibrinogen was statistically significant compared to HCs (*p* = 0.0445) and compared to SLE (*p* = 0.0069).

Because the stoichiometry of citrullination of untreated albumin is low (except for R98), we systematically screened RA and HC sera by ELISA for IgG binding to 14 citrullination sites using 11 pairs of peptides, 9 to 14 amino acid residues in length, with or without citrulline (Fig. 4*C*). Plates were coated with each unmodified (Arg-containing) peptide or its citrullinated version, incubated with 1:500 diluted serum, washed, and quantitated for bound IgG with an anti-human IgG secondary antibody. To focus on citrulline-specific antibodies we subtracted the unmodified peptide values from the citrullinated peptide values in each pair. Negative values were deemed to represent absence of anti-citrulline peptide reactivity. For full transparency, all raw values are shown in Fig. S2.

These measurements resulted in a range of absorbance values, with 38 to 80 of the RA samples yielding values > 0, *that is*, stronger binding to the citrullinated form of each peptide (Fig. 4*D*). However, this was also true for a similar proportion of serum samples from healthy donors (Fig. 4*E*). Using the 95th percentile of the healthy donor values for each individual peptide pair (indicated as dotted lines in Figure 4, *D* and *E*), we conclude that only very few RA patients had IgG noted as “positive” for the citrullinated sites in albumin: 28 RA sera (32.6%) were above the 95th percentile for at least one site. These are all summarized in Table 5. None of them reached statistical significance, except the citrullinated peptides corresponding to R114/117, R428, and R484/485, which were lower in RA than in healthy donors.

**Table 5:** IgG binding to citrullinated albumin peptides in RA (n = 86)

Site	Samples with >0^a^	≥ 95th percentile of HC
R81	52 (60.47%)	3 (3.49%)
R98	63 (73.26%)	6 (6.98%)
R114/117	38 (44.19%)	4 (4.65%)
R144/145	59 (68.60%)	4 (4.65%)
R218/222	44 (51.16%)	11 (12.79%)
R257	80 (93.02%)	7 (8.14%)
R410	45 (52.33%)	4 (4.65%)
R428	55 (63.95%)	1 (1.16%)
R445	47 (54.65%)	2 (2.33%)
R484/485	58 (67.44%)	1 (1.16%)
R521	62 (72.09%)	5 (5.81%)
TOTAL^b^	86 (100%)	28 (32.56%)

HC, healthy control; RA, rheumatoid arthritis.

a Higher IgG reactivity toward the citrullinated than the unmodified peptide.

b Note that this is not the sum of the above numbers because many patients had reactivity against more than one site.

Analyzing the seronegative and seropositive RA samples separately revealed very minor differences. The five patients with reactivity toward the citrullinated peptide containing R521 above the 95th percentile were all seropositive, while the three patients binding R81 were seronegative.

## Discussion

Our data reveal that albumin is citrullinated at multiple sites in healthy donors and hence must be a physiological event and not specific for RA. At first glance, this conclusion may appear to be in conflict with our 2019 paper (36), in which we reported that 38% of RA patients had IgG autoantibodies reacting with albumin citrullinated by PAD4 *in vitro*. However, we did not know that untreated albumin was already citrullinated and we were not able to determine if the bound IgG was specific for citrullinated albumin or merely represented cross-reactive ACPA. Our present study suggests that ACPA specific for individual citrullinated residues in albumin are, at best, very few.

Our findings support the notion that a state of T cell tolerance must exist for physiological citrullination and, importantly, that this tolerance appears to persist in RA patients. While it has been theorized that loss of tolerance toward citrullinated proteins underlies RA pathogenesis (6), we have proposed the alternative hypothesis that RA represents the consequences of T and B cell immunity against novel citrullinated epitopes against which preexisting tolerance never developed (11). Consequently, we distinguish between physiological citrullination, which occurs in all of us, *versus* pathological citrullination which is unique to patients developing RA and that entails the creation of “neoepitopes” for which immunological tolerance does not exist (11). As is the case with citrullinated albumin, ACPA in the serum of RA patients may cross-react with physiologically citrullinated proteins, which does not mean that these proteins were immunogenic themselves. Naturally, we cannot exclude the possibility that during the progression of RA epitope spreading may broaden to include physiologically citrullinated proteins. The relationship between citrullination and autoimmunity is likely even more complex. Replacing Arg with citrulline does not always enhance human leukocyte antigen allele DR binding and does not necessarily trigger a T cell response (37). Becart *et al.* (37) tested the human leukocyte antigen allele DR binding affinity and immunogenicity of four citrullinated peptides from aggrecan, vimentin, fibrinogen, and type II collagen and found that T cell responses to the four peptide pairs varied dramatically.

As albumin citrullination appears to be physiological, it has presumably evolved for some useful purpose. Although there is limited data on structural and/or functional changes resulting from citrullination, the few existing studies on albumin citrullination in RA synovial fluid (38, 39), non-RA synovial fluid (40), both healthy (41) and diseased tissues (42), and various animal albumins (43, 44, 45) have collectively reported all of the same citrullinated sites in included in the present study. However, most studies did not compare citrullination between groups (38, 39), and those that did reported no statistically significant differences between RA their non-RA control. Despite this, several informative studies have been published concerning other posttranslational modifications and mutations in albumin (Table 6). These alterations mimic the loss of charge, a key feature of citrullination, and may therefore incur similar consequences for the protein.

**Table 6:** Literature relevant to albumin citrullination sites

Site	Relevant interactions, mutations, and neighboring residues
R81	⁃ Critical for FcRn binding, forms hydrogen bond with T153 of FcRn (28) ⁃ Citrullination: Subtle, local elongation of preceding α-helix (D63-T76), geometry mostly preserved (46); effect on FcRn binding not reported. ⁃ *R81A:* FcRn binding reduced by 50% (29)
R98	⁃ Citrullination: conformational changes or changes in binding partners so that modified site is less accessible (51)
R114/117	⁃ Forms interdomain salt bridge with E520 (52) ⁃ *R114G*: decreased bilirubin binding (53), FcRn binding increased 1.8x (29)
R144/145	⁃ Nearby ligands: ruxolitinib, (54), thyroxine (55)
R218/222	⁃ Nearby ligands: methotrexate (48), thyroxine (56, 57), prednisone (55) ⁃ Stabilizes ligands but not necessary for binding geometry (58). Dictates steric constraints of binding site (57). ⁃ Citrullination: increased ligand binding affinity from loss of charge (59) ⁃ *R218H* (60, 61), *R218S* (62), *R218P* (63), *R218A* (64), *R222I* (65), *R222M* (66): increased thyroxine binding, increased total serum thyroxine.
R257	⁃ Nearby ligands: hydroxychloroquine (67), thyroxine (56, 57), paracetamol (68) ⁃ Stabilizes ligands by acting as proton acceptor during binding (58)
R410	⁃ Nearby ligands: NSAIDs (69, 70), chloroquine (67), thyroxine (56, 57) ⁃ *R410A*: decreased albumin T_1/2_ (47), decreased esterase activity (71)
R428	⁃ Lower pKa due to interactions with neighboring residues, more prone to modifications (70) ⁃ T422, E425: involved in FcRn binding (52)
R445	⁃ Nearby ligands: paracetamol (68) ⁃ *Q441A*: reduced FcRn binding (72)
R484/485	⁃ Nearby ligands: methotrexate (48), certain NSAIDs (73, 74) ⁃ *R485A*: reduced ketoprofen binding
R521	⁃ Citrullination: only observed in RA synovial fluid (39) ⁃ *K524*: interacts with FcRn during binding (52) ⁃ *K519E*: abolishes FcRn binding (30, 31) ⁃ I523G: increases FcRn binding ∼40-fold by introducing kink in helix under W53 of FcRn that improves fit (31)

FcRn, neonatal Fc receptor; NSAIDs, non-steroidal anti-inflammatory drugs.

Taldaev and colleagues (46) conducted a molecular dynamics simulation of numerous citrullinated proteins, including albumin. Specifically, this group investigated structural changes resulting from citrullination of albumin at R81 and found a slight elongation of the α-helix preceding the modification, presumably caused by the loss of the hydrogen bond between the guanidinium group of R81 and D89 that likely stabilize the albumin-FcRn interface (Fig. 2*A*). While the actual effect on FcRn binding induced by R81 citrullination is not known, it has been shown that mutations of R81 to alanine reduced the binding of albumin to FcRn by 50%, in agreement with the close approximation of R81 and its interaction with D89 to FcRn (Fig. 2*L*). Two additional Arg residues may also affect albumin binding to FcRn, namely R410 and R521. Mutation of R410 to alanine reduced the half-life of albumin (47), perhaps due to a change in FcRn binding (47), while mutations close to R521, such as K519E and I523G (31) abolished or enhanced FcRn binding, respectively. The side chain of K524 interacts directly with FcRn during binding. However, other residues may also affect this interaction despite lacking direct contact, for example, through complex interactions with other side chains that induce conformational alterations and alter binding affinity. Based on these considerations, one could speculate that albumin citrullination may serve to regulate its recirculation by FcRn and, hence, its half-life in circulation.

It has been reported that surface-expressed PAD4 on neutrophils increases in response to inflammatory stimuli and paralleled by a decrease in secreted PAD2 (32), suggesting the two enzymes are differently regulated and likely have some different functions, some of which may be reflected in the pronounced difference between PAD2 and PAD4 citrullination of R81. R81 citrullination might accelerate the clearance of albumin from circulation, potentially explaining the commonly reported low albumin levels in RA and other inflammatory conditions. Thus, it seems plausible that this site in particular could be a crucial mediator of albumin degradation, perhaps in response to inflammation. The physiological implications of R81 citrullination, as well as citrullination at other sites, should be further explored.

Another potential physiological role of albumin citrullination may be related to the location of several citrullinated Arg residues close to the six ligand binding pockets of albumin. Indeed, several mutations of Arg residues, such as R114, R218, R222, R257, R410, and R428, influence ligand binding. Our data also support the notion that albumin citrullination reduced the binding of T4 (Fig. 3*D*). Thus, the binding and release of hormones, metabolites, fatty acids, or drugs may be regulated by albumin citrullination in a manner that may depend on location and time in circulation given that citrullination is irreversible.

Nine of the 11 RA patients with reactivity against the peptide corresponding to citrullinated R218/R222 above the 95th percentile reported current methotrexate use. Compared to all other patients on methotrexate (n = 47), the patients with this reactivity showed a negative correlation between methotrexate dose and immunoreactivity at this site. Given that methotrexate binds to site I and R222 acts as a proton donor to form two of five hydrogen bonds with methotrexate in this site (48), it is possible that citrullination of R222 (and perhaps R218) may reduce methotrexate binding. If so, the concentration of free drug may be increased resulting in increased pharmacological effects of methotrexate. However, the 0 to 9.68% stoichiometry of citrullination at this site (Table 3) leaves the relevance of such an effect questionable.

Taken together, our findings support the notion that citrullination of albumin is part of normal physiology and not restricted to RA. It does not appear the citrulline-directed immune response in RA is targeting citrullinated albumin; the few weakly binding IgGs that barely cross over the 95th percentile of the healthy donor distribution are unlikely to be truly selective for citrullinated albumin. More likely they represent ACPA against other citrullinated proteins that have some modest degree of sequence similarity to the Arg residues citrullinated in albumin. Only one RA patient had IgG binding to purified albumin (Fig. 4*A*), but this same patient did not recognize any of the citrullinated peptides, suggesting that this antibody was not even citrulline-specific. Indeed, anti-(unmodified?)albumin antibodies have been observed in a subset of SLE (49) or autoimmune bullous skin disease (50) patients. Even so, better characterizing the disease-specific repertoire of citrullinated proteins (“citrullinome”) in RA, with emphasis on identifying specific Arg residues and their citrullination stoichiometries, might elucidate more precise mechanisms behind the pathogenesis of this disorder.

## Experimental procedures

### Patients

Sera from RA patients (n = 86), SLE patients (n = 24), and HCs (n = 33) were from the UW Rheumatology Biorepository and kept at −20 °C until use. Only sera collected within the last 4 years were used to minimize the risk of sample degradation. The patient characteristics for this cohort are summarized in Table 1. Synovial fluid from two RA patients, one healthy individual, and one juvenile idiopathic arthritis patient were from the same resource. Freshly drawn blood from 4 RA patients was used for neutrophil isolation. Institutional Review Board approval for our study was obtained from the University of Washington ethics board for the Biorepository (STUDY00003007) and our study (STUDY00006196), and informed written consent was obtained from all participants according to the Declaration of Helsinki.

### Peptides and reagents

Eleven pairs of 9 to 14 amino acid residue peptides (custom-ordered from Shanghai RoyoBiotech Co.) were used, each pair consisting of the unmodified peptide with a central Arg and its citrullinated version (sequences shown in Fig. 4*C*). Horse radish peroxidase (HRP)-conjugated goat anti-human IgG was from ImmunoReagents, 3,3′,5,5′-tetramethylbenzidine (TMB) from BioLegend (#50-169-261), in-gel tryptic digestion kit from Thermo Fisher Scientific (#89871X), EasyPep in-solution tryptic digestion kit from Thermo Fisher Scientific (#A40006), 96% purified HSA from Thermo Fisher Scientific (#J66780.03), human AB Rh+ serum, heat-inactivated at 56 °C, from Atlanta Biologicals (now R&D Systems) (#S40110H), recombinant PAD2 and PAD4 (Cayman), Pierce protein G agarose beads from Thermo Fisher Scientific (#20397), Anti-Citrulline (Modified) Detection Kit from Sigma-Aldrich (#17-346B-1), Thyroxine Competitive ELISA kit from Invitrogen/Thermo Fisher Scientific (#EIATAC), and IMMUNOSCAN CCPlus from Svar Life Science AB (RA-96PLUS).

### Neutrophil isolation

Neutrophils were isolated from venous blood by gradient centrifugation on PolymorphPrep (CosmoBio) according to the manufacturer's instructions. They were subsequently washed and suspended in Dulbecco's modified Eagle's medium high glucose (#11-965-118, Gibco, Thermo Fisher Scientific).

### *In vitro* citrullination of albumin by PAD2 and PAD4

Two different sources of albumin, 96% purified HSA (Thermo Fisher Scientific) and AB+ serum (Sigma-Aldrich), first treated with protein G to ensure IgG depletion (see below section), were separately incubated with or without recombinant PAD2 and/or PAD4 in tris-buffered saline, 5 mM Ca^2+^, and 1 mM DTT. The 96% purified HSA was additionally treated with live RA neutrophils to compare with citrullination induced by recombinant PAD enzymes. After a 1 h incubation at 37 °C, samples were prepared for SDS-PAGE and LC-MS/MS analysis as described below.

### Synovial fluid sample preparation

Synovial fluid samples (n = 2 RA, n = 2 non-RA) and normal human AB+ sera were centrifuged, and supernatants were collected for further processing. Protein G agarose (Thermo Fisher Scientific) beads were added to all samples and incubated at 4 °C for 2 h with rotation to ensure IgG depletion. The unbound protein was collected.

One synovial fluid sample was subjected to an additional processing step: cold acetone (−20 °C) was added and incubated at −20 °C for 1 h. After centrifugation, the pellet was dried and then redissolved in PBS.

### In-gel trypsin digestion

Albumin in serum was resolved by SDS-PAGE and Coomassie blue staining and excised. Gel pieces were subjected to in-gel tryptic digestion per the manufacturer’s protocol (Thermo Fisher Scientific, #89871X). Briefly, they were destained with a destaining solution containing NH_4_CO_3_, reduced with a Tris-(2-carboxyethyl)phosphine hydrochloride (Thermo Fisher Scientific, #T2556) solution at 60 °C for 10 min, alkylated with iodoacetamide at room temperature for 1 h in the dark, and washed again with destaining solution. After shrinking gel pieces with acetonitrile, trypsin and digestion buffer were added and incubated overnight. Digestion mixtures were added to C-18 spin columns (Pierce/Thermo Fisher Scientific #89870) to further purify samples in preparation for mass spectrometry.

### In-solution trypsin digestion

Serum samples and purified albumin were alternatively digested in-solution per the manufacturer’s protocol (Thermo Fisher Scientific, #1863443). Briefly, samples were diluted to 0.1 to 1 mg/ml in lysis buffer, reduced with Tris-(2-carboxyethyl)phosphine hydrochloride (Thermo Fisher Scientific, #1863445) and alkylated with 2-chloroacetamide (Thermo Fisher Scientific, #1863446) at 95 °C for 10 min, and digested with a trypsin/Lys-C protease mix at 37 °C for 3 h. The digestion reaction was stopped with a formic acid–based digestion stop solution from the EasyPep kit (Thermo Fisher Scientific, #1863443) and samples applied to the provided sample clean-up columns.

### AMC blot to assess albumin citrullination

Purified albumin (Thermo Fisher Scientific) was treated without or with PAD4 and then resolved by SDS-PAGE and transferred to a polyvinylidene fluoride membrane. Modification buffer from the AMC detection kit was added to the blot and incubated overnight at 37 °C. Blocking was with 5% nonfat milk diluted in tris-buffered saline with 0.2% Tween-20 (TBST) for 1 h. AMC antibody was diluted 1:1000 in milk-TBST, incubated for 2 h, and washed with TBST. 1:2000 goat anti-human HRP in milk-TBST was added for a final 1-h incubation and wash. Finally, signal detection was achieved using Luminata Crescendo Western HRP substrate and the blot was imaged using the Gel-Doc imaging system.

### Liquid chromatography-tandem mass spectrometry

Purified samples stored at −80 °C were thawed right before mass spectrometry analysis and 0.1% formic acid added to resuspend peptides and injected into an Orbitrap Exploris 480 (University of Washington’s Proteomics Resource).

All samples were analyzed on an Exploris480 mass spectrometer (Thermo Fisher Scientific) equipped with an EASYnLC 1200 UPLC system (Thermo Fisher Scientific) and in house developed nano spray ionization source. One microliter samples were loaded from the autosampler onto a 100 μm ID IntegraFrit trap (New Objective) packed with Reprosil-Pur C18-AQ 120 Å 5 μm material to a bed length of 3 cm with a volume of 18 μl at a flow rate of 2.5 μl/min. After loading and desalting with 0.1% formic acid in water (LC-MS grade from Fisher), the trap was brought in-line with a pulled fused-silica capillary tip (75 μm i.d.) packed with 35 cm of Reprosil-Pur C18-AQ 120 Å 5 μm (Dr Maisch). Peptides were separated using a linear gradient, from 6 to 35% solvent B (LC-MS grade 0.1% formic acid, 80% acetonitrile in water (Fisher)) in 120 min at a flow rate of 300 nl/min. The column temperature was maintained at a constant 50 ^o^C during all experiments.

Peptides were detected using a data-dependent method. Survey scans of peptide precursors were performed in the orbitrap mass analyzer from 375 to 1575 *m/z* at 120 K resolution (at 400 *m/z*) with 300% normalized Automatic Gain Control target and a maximum injection time of 25 ms. After the survey scan, tandem mass scanning was performed for 1 s on the most abundant precursors exhibiting a charge state from two to five of greater than 1e4 intensity by isolating them in the quadrupole with an isolation width of 1.6 *m/z*. Higher energy collisional dissociation fragmentation was applied with a normalized collision energy of 30%. Resulting fragments were detected in the orbitrap mass analyzer at 15 K resolution (at 400 *m/z*) with a 300% normalized Automatic Gain Control target and a maximum injection time of 40 ms. The dynamic exclusion was set to 30 s and isotopes were excluded. In addition to automatic filtering from the database search, the data were also manually filtered to ensure proper identification of citrullinated peptides. Only entries with a corresponding expect value < 10^-4^ and probability >0.75 were considered for analysis. Citrullinated peptides were detected based on the mass change of Arg to 157.09 Da. Peptides containing citrulline as the last residue were assumed to be false positives due to trypsin’s inability to cleave after citrulline and were thus excluded from analysis.

### Database searches and citrullination stoichiometry

Thermo.raw files were converted to the mzXML format using the ReAdW (version 2016.1.0) converter. The mzXML files were searched against a Species RAT.fasta protein sequence database downloaded from UniProt appended with common contaminant sequences (24,000 total sequence entries). A database search was performed using Comet (2021.02 rev. 0 (3c62af2)) with the following search parameters: 20 ppm precursor tolerance, concatenated target-decoy search, tryptic digest allowing two missed cleavages, variable modifications for oxidized methionine and phosphorylation on STY, and static modification for carboxyamidomethylation on cysteine. The Comet search results were then processed with PeptideProphet and ProteinProphet tools from the Trans-Proteomic Pipeline software suite (TPP v5.0.0 Typhoon; http://tools.proteomecenter.org/wiki/index.php?title=software:TPP#Mac_OSX).

For each citrullinated Arg, the % citrullination was calculated from the sum of all peptides with that citrullinated Arg divided by the total peptides with that same Arg (both unmodified and citrullinated). An overall % citrullination was also computed from the sum of all citrullinated peptides divided by all Arg-containing peptides. Additional details and the raw data used for these citrullination calculations, as well as specific details on patient characteristics and disease parameters for each sample, can be found in File S1.

### Enzyme-linked immunosorbent assay

Ninety-six–well polystyrene plates were coated with 200 ng/well of each peptide in a 0.1 M carbonate (pH 9.6) buffer overnight. Plates were washed three times with PBS with 0.05% Tween-20 (PBST) and blocked with 10% nonfat milk in PBST for 2 h. After washing with PBST four times, patient sera were diluted 1:500 in 10% nonfat milk and incubated for 2 h before four washes with PBST. One hundred microliters of 1:2000 diluted secondary antibody, HRP-conjugated goat anti-human IgG, in 10% nonfat milk was added to each well. After 45 min, five washes with PBST were performed before adding 100 μl TMB substrate to each well and incubating in the dark for approximately 10 min. Fifty microliters H_2_SO_4_ was then added to each well to stop the color reaction and plates were immediately read at 450 nm and calibrated at 630 nm using a plate reader. All reagents used were freshly prepared immediately prior to use and allowed to warm to room temperature. All plates were repeated at least three times to control for experimental variability and final values were averaged from all runs. The absorbance values obtained with each unmodified peptide were subtracted from the values with the corresponding citrullinated peptide, and positive values were considered to indicate higher IgG binding of the citrullinated peptide (cit > R). Negative values were interpreted as lack of citrulline selectivity (*i.e.*, higher IgG binding to the unmodified peptide) and were thus set to 0 for subsequent data analysis. For each peptide, a cutoff corresponding to the 95th percentile of the HC cohort was calculated and only values above this threshold were deemed positive.

### CCP assay

To validate the CCP status given in patient records (some of them several years old) and to cover for missing information, a semiquantitative protocol from the IMMUNOSCAN CCPlus kit was used to determine IgG antibodies to CCP in RA sera. According to the manufacturer’s protocol, briefly, 100 μl of controls and calibrators (dilutions of CCP + sera with known values) were added in duplicates to a 96-well plate precoated with synthetic CCP peptides, followed by 100 μl of RA sera diluted 1:50 in dilution buffer (provided by the kit). The plate was incubated for 1 h before rinsing 3 times with 300 μl wash buffer (provided by the kit). One hundred microliters anti-IgG–HRP conjugate solution was added to each well for an additional 30 min incubation. After another three washes with the same volume of wash buffer, 100 μl TMB substrate solution was added to all wells and incubated for another 30 min, after which 100 μl 0.5 M H_2_SO_4_ was added to stop the color reaction. The plate was then read at 450 nm within 10 min. All reactions were carried out at room temperature. For data interpretation, the average negative control value was first subtracted from all other wells. A 4PL equation was derived in the same manner as in the thyroxine assay below and used to transform raw absorbance values into anti-CCP antibody titers. Samples <25 U/ml were defined as negative (CCP-) and those >25 U/ml were considered positive (CCP+).

### Assay for thyroxine binding to albumin and citrullinated albumin

Thirty micrograms of purified albumin was incubated with or without 50 ng PAD4 in 150 mM NaCl, 25 mM Tris/HCl, pH7.8, with 5 mM Ca^2+^, and 1 mM DTT at 37 °C for 30 min. One nanogram of T4 thyroxine was added for an additional 30 min incubation at 37 °C and then transferred to a Centricon-10 spin column (10 kDa cutoff) to separate free thyroxine from thyroxine bound to albumin. Dissociation reagent was then added to the remaining albumin solution (above the filter), incubated at room temperature for 5 min, and diluted 1:20 in assay buffer and subjected in triplicates to a T4 quantitation ELISA kit according to the manufacturer’s (Invitrogen/Thermo Fisher Scientific) instructions together with a thyroxine standard dose range of 20, 10, 5, 2.5, 1.25, 0.625, and 0 ng/ml, which was used to create a four parameter logistic (4PL) standard curve equation from an online graphing tool (AAT Bioquest, 2024, Quest Graph Four Parameter Logistic (4PL) Curve Calculator). Data points were transformed using the 4PL equation and multiplied by the dilution factor to obtain final T4 values.

### Statistics

The Mann–Whitney *U* test was used to compare values obtained with RA samples to those from healthy donors. As a cutoff for antibody positivity, we used the 95th percentile of the HC values. GraphPad Prism (https://www.graphpad.com/features) was used for the analyses, which were considered statistically significant at *p* < 0.05.

## Data availability

The raw mass spectrometry data reported in this study can be accessed through PRIDE ARCHIVE *via* [TBD].

## Supporting information

This article contains supporting information.

## Conflict of interests

Tomas Mustelin reports a relationship with Cugene Therapeutics, Miro Bio, ROME Therapeutics, Evolved, and Codify Therapeutics that includes: consulting or advisory. The other authors declare that they have no conflicts of interest with the contents of this article.
