# Supplementary material for: Extensive citrullination of human serum albumin is physiological and not inherently immunogenic in rheumatoid arthritis
Source: J Biol Chem. 2025 Jul 1;301(8):110438. doi: 10.1016/j.jbc.2025.110438 (PMC12314391; doi:10.1016/j.jbc.2025.110438)

Supplemental data:

Fig. S1. Representative mass spectra of albumin peptides with citrullinated Arg residues. The 6 spectra contain the indicated citrullination site and come from either an RA patient (RA) or a healthy control (HC), as indicated.

Fig. S2. ELISA data for IgG binding to the same peptides as in Fig. 4D and E. Top panels, ELISA results for the unmodified peptides. Middle panels, ELISA results for the citrullinated peptides. Bottom panels, the same data with reactivity against the unmodified peptide subtracted from the reactivity against the citrullinated peptide. Left side panels, RA patient (n=87), right hand panels, healthy controls.

Supplemental Fig. S1.

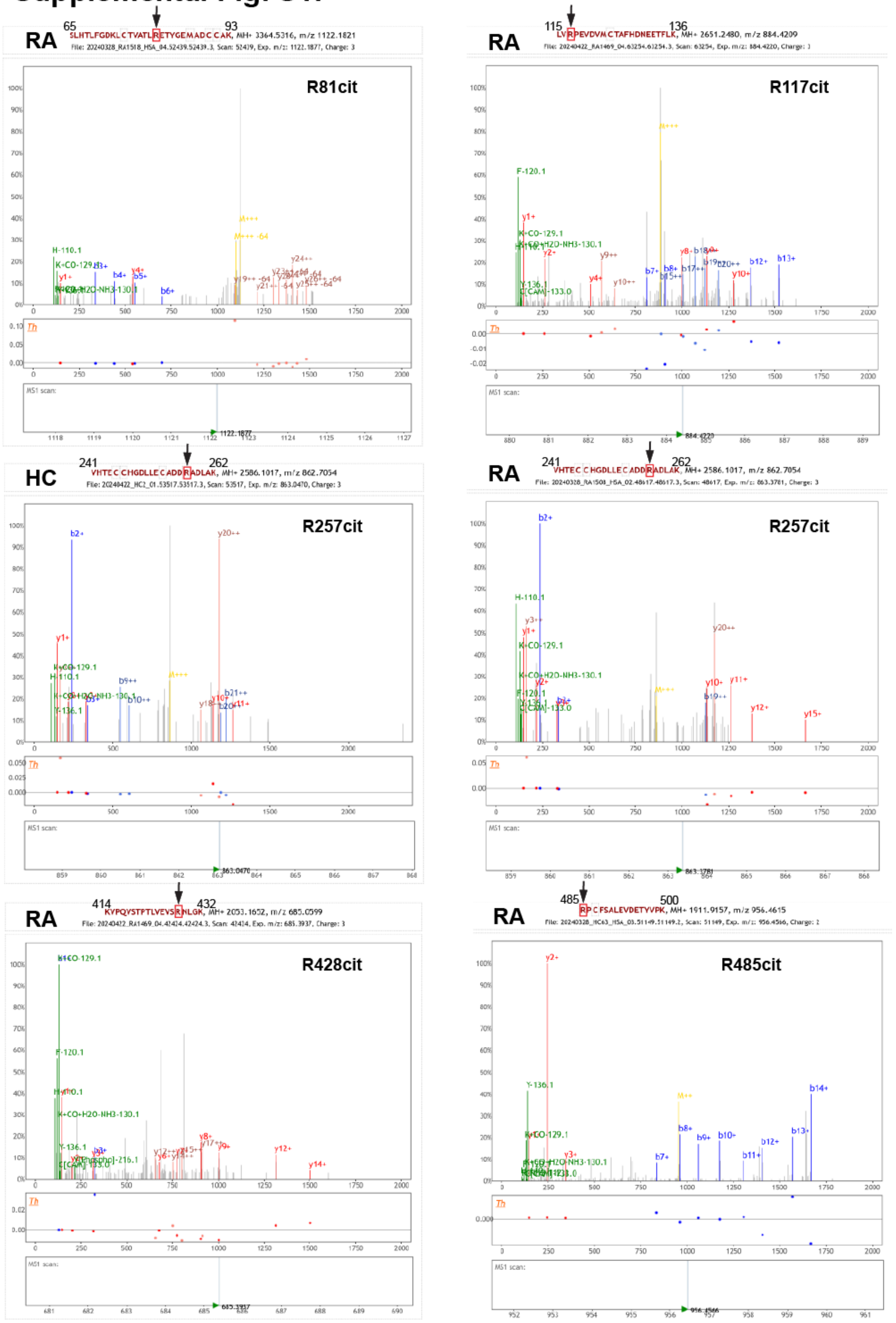

Supplemental Fig. S2.

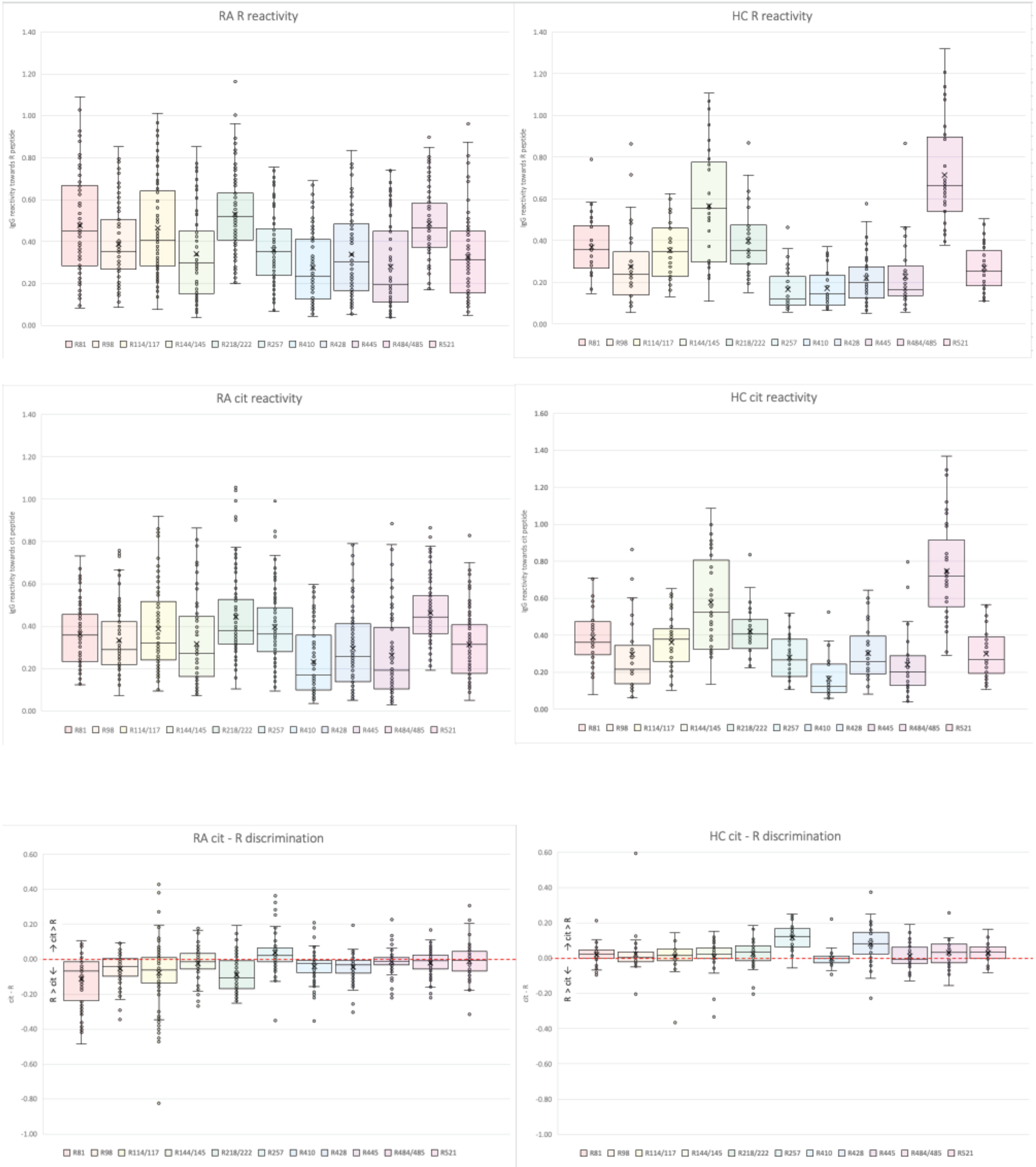

Supplement: Supplemental material [file mmc2.pdf]
